# Beware ‘persuasive communication devices’ when writing and reading scientific articles

**DOI:** 10.7554/eLife.88654

**Published:** 2023-05-25

**Authors:** Olivier Corneille, Jo Havemann, Emma L Henderson, Hans IJzerman, Ian Hussey, Jean-Jacques Orban de Xivry, Lee Jussim, Nicholas P Holmes, Artur Pilacinski, Brice Beffara, Harriet Carroll, Nicholas Otieno Outa, Peter Lush, Leon D Lotter

**Affiliations:** 1 UCLouvain Louvain-la-Neuve Belgium; 2 Access 2 Perspectives Berlin Germany; 3 University of Surrey Guildford United Kingdom; 4 Université Grenoble Alpes Grenoble France; 5 Institut Universitaire de France Paris France; 6 Ruhr University Bochum Bochum Germany; 7 KU Leuven Leuven Belgium; 8 Rutgers University New Brunswick United States; 9 University of Birmingham Birmingham United Kingdom; 10 Université d’Angers Angers France; 11 Université de Nantes Nantes France; 12 Lund University Lund Sweden; 13 University of Aberdeen Aberdeen United Kingdom; 14 NHS Grampian Aberdeen United Kingdom; 15 Maseno University Kisumu Kenya; 16 University of Sussex Brighton United Kingdom; 17 Research Center Jülich Juelich Germany

**Keywords:** scientific writing, scientific publishing, citation, reporting, language, point of view, Human

## Abstract

Authors rely on a range of devices and techniques to attract and maintain the interest of readers, and to convince them of the merits of the author’s point of view. However, when writing a scientific article, authors must use these ‘persuasive communication devices’ carefully. In particular, they must be explicit about the limitations of their work, avoid obfuscation, and resist the temptation to oversell their results. Here we discuss a list of persuasive communication devices and we encourage authors, as well as reviewers and editors, to think carefully about their use.

Writing a research article is difficult for many reasons. First, there are many things that need to be done besides writing, such as generating the figures. Second, a research article is typically several thousand words long, so there is a lot of writing to do and many decisions to make (such as what to include and what to leave out). Third, most articles have multiple authors, so it will be necessary to circulate drafts to co-authors and act on their feedback. In this article we will focus on the second of these tasks – the act of writing the article itself.

All writing that is intended for publication should be clear and engaging, and the authors of scientific articles can use a wide range of persuasive communication devices to achieve these goals. An obvious example is to give the article an eye-catching title, and this is perfectly fine if the title reflects the content of the paper. However, it is also possible for these devices to be mis-used in ways that can mislead readers (including reviewers) by, for example, giving a false impression about the significance of the work being reported. Clearly, this is not fine.

In this article – which builds on a preprint we posted in March 2022 (Corneille et al., 2022) – we describe a range of persuasive communication devices that can be used to exaggerate the importance of and/or hide the weaknesses of scientific work, and urge authors to exercise caution when thinking about using such devices.

## A tentative typology of persuasive communication devices

Good scientific writing is hard, and it is easy to make mistakes, so we will not point to examples in the literature. Instead, over the last year, we have reflected on our own writing styles, and on the writing styles we have encountered when reading or reviewing academic articles, chapters and books. We have shared these reflections with each other and compiled a list of persuasive communication devices, which we have organized in a tentative typology (Table 1). This typology contains 22 devices arranged in four categories: mischaracterizing the state-of-the-art; overselling; smoke screening and deflection; and the misuse of authority (and authors). We hope that this list will encourage reflection on, and fostering of, good scientific writing.

**Table 1.** Persuasive communication devices.

	Device	Description
**Category: Mischaracterizing the state-of-the-art**		
**1**	Ignoring previous work	Not citing previous work that decreases the perceived novelty of the current work.
**2**	One-sided citation	Mostly or only citing supportive research, and mostly or completely ignoring research that does not support the author’s point of view.
**3**	Reliance on weak evidence	Citing work that is now known to be weak or wrong.
**4**	Misleading use of references	Citing papers that are not relevant to the point the author is trying to make in order to give the impression that support for this point is stronger than it actually is.
**5**	Missing evidence	Making statements that are not backed up with citations.
**Category: Overselling**		
**6**	Excessive titles	Using titles which make claims that go beyond the findings being reported.
**7**	Overgeneralization	Generalizing results beyond the population studied without evidence to support such claims.
**8**	Hype	Using adjectives such as striking, important, remarkable and so on without justification.
**9**	Selective reporting	Not reporting findings that would make the article ‘weaker‘; not reporting hypotheses that have been tested and ruled out.
**10**	Hypothesizing after the results are known (HARKing).	Giving the impression that a hypothesis was formulated before data were collected, when it was formulated after data collection.
**Category: Smoke screening and deflection**		
**11**	Inconsistent terminology	Being inconsistent in the use of terminology across papers – and sometimes within a paper –in order to avoid scrutiny.
**12**	Selective quotation	Selectively quoting other work, or citing other work out of context, in order to make a point.
**13**	Straw-person argument	Exaggerating or distorting other work in order to easily refute it.
**14**	Cryptic writing	Writing in a way intended to make an article unnecessarily difficult for readers to understand in order to impress them and prevent a fair assessment of the work being reported.
**15**	(Supplementary) information overload	Overwhelming the reader with poorly organized supporting materials, if done to prevent close scrutiny.
**16**	Limiting what is said about limitations	Seeking to downplay or hide the limitations of a study.
**17**	Ambiguity	Using words which suggest more than what the study delivered.
**18**	Selective appeal for rigor	Requiring higher standards of evidence from researchers with a different or competing perspective.
**19**	Open research washing	Engaging in ‘open research‘ practices in a superficial manner in order to boost the perceived robustness of work.
**Category: Misuse of authority (and authors**)		
**20**	Reliance on precedent	Suggesting that a procedure with known flaws is suitable for a study because it has been used in lots of previous studies.
**21**	Reliance on number of citations	Arguing that because previous work has received lots of citations, an area of research – and hence the current work – is important and of high quality.
**22**	Honorary authorship	Including a well-known researcher in the author list – even though they do not meet the relevant criteria for being an author – in order to increase the chances of the manuscript being accepted for publication.

### Mischaracterizing the state-of-the-art

Researchers are at risk of misleading the reader about the novelty or strength of their research when they make inaccurate statements about the current state of scientific knowledge. This mischaracterization can take various forms and often involves failing to cite articles that are relevant, or citing articles that are not relevant. For example, authors might fail to cite articles that have already reported a similar result (device 1 in Table 1; ignoring previous work), ignore work that is inconsistent with their own work (device 2; one-sided citation), cite supportive work of weak quality (device 3; reliance on weak evidence), cite work that is agnostic to the point the author is trying to make (device 4; misleading use of references), or make statements that are not backed-up by a relevant citation (device 5; missing evidence). It is important to avoid bias in the reporting and citing of previous research because the cumulative effects of such biases can, for example, inflate the apparent efficacy of medical treatments (see de Vries et al., 2018 for a study of treatments for depression).

It is probably not possible to estimate how often authors fail to cite articles they should have cited (devices 1 and 2). However, as regards articles that are cited, it has been estimated that around a quarter of the articles cited in ‘high-impact general science journals‘ do not completely support the statement they are supposed to support (Smith and Cumberledge, 2020), and similar findings have been reported in psychology (Cobb et al., 2023). Another problem is the practice of citing an original study without citing failures to replicate it (von Hippel, 2022): indeed, it has been estimated that only 12% of citations of non-replicated findings acknowledge the failure(s) to replicate (Serra-Garcia and Gneezy, 2021). However, it should also be noted that authors are sometimes compelled to cite articles that are not relevant by editors hoping to increase the impact factor of their journal (Fong et al., 2023).

### Overselling

The second category in our typology concerns authors trying to inflate the perceived importance of their own research. This can involve the use of attention-grabbing titles which make claims that go well beyond the findings of a study (device 6; excessive titles), or authors overgeneralizing their conclusions without sufficient evidence (device 7; overgeneralization). Examples of the latter might include generalizing from college students to all human adults, or from one species to another. The inappropriate use of adjectives such as ‘striking,‘ ‘important,‘ or ‘remarkable,‘ and wording that makes the author’s research questions seem more important than they really are, are also problematic (device 8; hype). Reviewers will often ask authors to tone down their language, but one journal (*ACS Catalysis*) has gone a step further and now pre-screens submissions for words like ‘outstanding,‘ ‘excellent,‘ and ‘unprecedented‘ (Scott and Jones, 2017). The hyping of research has been particularly noticeable during the COVID-19 pandemic (Hyland and Jiang, 2021).

The perceived coherence of the narrative can also be increased by not reporting findings that would weaken the article (device 9; selective reporting), or by claiming that the results were consistent with a hypothesis when, in fact, the hypothesis was generated after the data had been collected (device 10; HARKing). The practice of HARKing (which is short for hypothesizing after the results are known; Kerr, 1998), has been widely discussed in the literature (for a nuanced discussion, see Hollenbeck and Wright, 2017 and Rubin, 2017).

### Smoke screening and deflection

The third category contains devices that are designed to reduce transparency and prevent debate. One tactic is being inconsistent in the use of terminology in order to prevent other researchers testing – and possibly refuting – an author’s claims (device 11; inconsistent terminology): in psychology, for example, an author might describe the same mental process as efficient in one article, unconscious in a second article, and unintentional in a third. Another tactic is to mislead the reader about other work being cited through selective quotation, misquotation, or quotation out of context (device 12; selective quotation).

Relying on ‘straw-person‘ arguments is another form of smoke screening: an author may interpret a claim or theory in its most exaggerated form, and then try to convince readers who are not familiar with the nuances of the claim or theory that it is foolish and therefore wrong (device 13; straw-person argument: see, for example, Aikin and Casey, 2022). Likewise, writing that is cryptic, obscure or undecipherable can convey a false sense of expertise and may prevent a fair assessment of the claims, theories, or analyses in a paper (device 14; cryptic writing: see, for example, Frankfurt, 2005). In a related tactic, an author may seek to overwhelm the reader with large volumes of poorly structured/explained supplementary material in order to prevent scrutiny of a paper’s conclusions (device 15; (supplementary) information overload). Smoke screening can also be implemented by not noting all the limitations of a study, or highlighting relatively minor limitations while seeking to downplay more serious limitations (device 16; limiting what is said about limitations).

Authors can also use ambiguous or polysemous terminology to inflate claims or deflect critiques (device 17; ambiguity). For instance, the word ‘influence‘ can be used to suggest causality, without explicitly claiming that causality has been demonstrated, thus providing the author with wiggle room if another researcher challenges the finding. Words like ‘influence‘ are particularly deceptive when used to describe results derived from weak theories where causality cannot be easily identified (Rohrer, 2018). Another way for an author to deflect a critique is to demand stronger evidence from a researcher with a competing perspective than the author demands from themself (device 18; selective appeal for rigor).

Superficial engagement with open research practices (such as underspecifying a pre-registered study) can also be used to mislead readers and reviewers about the strength of research (device 19; open research washing). For instance, one study of data availability in economics found that only 47.5% of articles in journals with a data-availability policy actually complied with the policy (Vlaeminck and Podkrajac, 2017; see also Tedersoo et al., 2021).

### Misuse of authority (and authors)

Our fourth category is a range of devices that rely on authority rather than sound arguments. An author may suggest, for example, that some procedure (e.g., a measurement technique) is valid because it has been used in a large number of studies, even though the procedure is known to have flaws (device 20; reliance on precedent). Likewise, an author may write that there is growing interest in X or that there have been lots of papers about Y to give the impression that their own paper is more important than it really is (device 21; reliance on number of citations).

Honorary authorship is the practice of including someone as an author on a paper because they are famous and/or important, even though their contribution to the article in question does not warrant their inclusion in the author list (device 22; honorary authorship). Honorary authorship is a well-known problem in medicine (Flanagin et al., 1998; Macdonald, 2022).

## Where do we go from here?

Our intention in writing this article was to recognize how difficult it is to effectively and accurately convey one’s research to the scientific community and beyond, while at the same time encouraging self-reflection amongst authors, reviewers and editors. This self-reflection should focus on the potential misuse of persuasive communication devices in scientific writing, so that as a global scholarly community we can uphold the highest possible standards of research rigor with a level-playing field.

We want to emphasize that the issues listed here do not necessarily arise from a deliberate intention on the part of the author to mislead the reader. For instance, ignorance of relevant work need not be deliberate, or there may be a limit on the number of references that can be cited. More generally, authors may simply conform to examples set in other papers. However, we find it useful to raise awareness of writing practices that may lead to the misinterpretation of research results, both within and outside our scientific community. Moreover, the scientific writing issues discussed here can be detrimental even when they are not implemented purposefully.

The typology we present is tentative, and is not intended to be a dogmatic list of dos and don’ts. We do not mean to suggest either that incoherent narratives or exceedingly boring or technical titles and abstracts should be the rule. However, there is a point at which the writing style of an article, chapter, or book, is at risk of misleading the readers, be it on purpose or not. In its extreme version, an article may ‘camouflage true phenomena in the name of promoting Wow Effects and preferred narratives‘ (Jussim et al., 2016).

Going beyond scientific writing per se, one should keep in mind that we may be tempted to use the same or related persuasive communication devices in all kinds of science communication. In oral talks, scientific poster presentations, interviews, and even in discussions with colleagues, we may find ourselves resorting to persuasive tactics that are not always consistent with good research practices.

The descriptions of the persuasive communication devices we have identified are necessarily brief as there are 22 of them overall. We are also certain that this is an incomplete list (see, for example, O’Donohue, 2023, for a discussion of the use of rhetoric in clinical science). However, we firmly believe that highlighting these issues and encouraging discussion around them is part of the solution. As authors prepare articles for submission, and as reviewers and editors assess these articles, we encourage them to think about the devices listed in Table 1, and to identify other devices that also have the effect of exaggerating importance or hiding weaknesses. In particular, we urge authors to reflect on whether any article they are writing or reviewing (formally or informally) is fair, and will bring readers closer to truth, or is just as likely to steer them away from it.
